# Tailored antiplatelet therapy can overcome clopidogrel and aspirin resistance - The BOchum CLopidogrel and Aspirin Plan (BOCLA-Plan) to improve antiplatelet therapy

**DOI:** 10.1186/1741-7015-9-3

**Published:** 2011-01-12

**Authors:** Horst Neubauer, Andreas FC Kaiser, Heinz G Endres, Jan C Krüger, Andreas Engelhardt, Sebastian Lask, Fenena Pepinghege, Andreas Kusber, Andreas Mügge

**Affiliations:** 1 Cardiovascular Center, Ruhr University Bochum, St. Josef Hospital, Gudrunstr. 56, D-44791 Bochum, Germany; 2 Department of Medical Informatics, Biometry and Epidemiology, Ruhr University Bochum, Overbergstr. 17, D-44801 Bochum, Germany

## Abstract

**Background:**

Dual antiplatelet therapy using acetylsalicylic acid (ASA, aspirin) and clopidogrel is of great importance following coronary stenting. However, the variable platelet inhibitory effectiveness compromises the antithrombotic advantages provided by dual antiplatelet therapy. The aim of this single-center prospective study was to reduce the low response incidence of dual antiplatelet therapy with ASA and clopidogrel according to a prespecified therapy algorithm.

**Methods:**

Platelet function testing using whole blood aggregometry (Chronolog 590) was performed 48 hours following coronary stenting (for either acute coronary syndromes or stable coronary artery disease) on 504 patients. The antiplatelet therapy included a loading dose of 600 mg clopidogrel and 500 mg ASA, followed by 75 mg clopidogrel and 100 mg ASA once daily. Clopidogrel low responders (CLR: >5 ohm; adenosine diphosphate (ADP) 5 μM) and/or ASA low responders (ALR: >0 ohm; arachidonic acid 10 μM) were treated according to a structured therapy plan: in the case of CLR, the maintenance + dose was doubled (repeated loading dose followed by 150 mg daily), and when still ineffective ticlopidine or prasugrel, if available and not contraindicated, were used. ALR was treated by increasing the dose to 300 mg in a first step or to 500 mg ASA when the first modification did not take effect sufficiently. In addition, ADP receptor antagonist 2-methylthioadenosine 5'-monophosphate triethylammonium salt (MeSAMP) testing and ASA incubation were performed to rule out either a platelet ADP-receptor defect or an ASA pharmacokinetic resistance.

**Results:**

Of the total cohort of 504 patients, we detected 30.8% clopidogrel low-responders and 19.4% aspirin low-responders. For ALR, with a dose adjustment of 300 mg ASA daily, 94.6% of ALR were effectively treated and the residual 5.4% by administration of daily dosages of 500 mg ASA. This means that after modification of the ASA maintenance dose, all initial ALRs had an adequate antiplatelet response.

The results for clopidogrel revealed that 69% of the CLR were treated effectively by increasing the clopidogrel dose to 150 mg daily. When prasugrel was not available or contraindicated, 12.7% of the remaining low responders showed an adequate result after being switched to ticlopidine. Consequently, by applying the therapy algorithm, we were able to reduce the CLR prevalence by 86.6%. On including prasugrel in the therapy plan, we were finally able to eliminate thienopyridine low response. In addition, no ADP receptor defect was found in this study as a potential reason for CLR.

We identified the following factors associated with both CLR and ALR status: acute coronary syndromes, positive troponin values as well as diabetes mellitus and elevated HbA_1C _values and a higher platelet count. Furthermore, our data revealed for CLR elevated C-reactive protein values and a high PREDICT-score (including an age >65 years, acute coronary syndrome, diabetes mellitus, renal failure, and reduced left ventricular function) as risk factors. The following factors correlated with the risk of ASA low response: patients with elevated hemoglobin, serum creatinine and C-reactive protein values. In addition, medication with nitrates reduced the risk of being CLR. As also holds true for CLR, we found the PREDICT-score to be correlated to the risk of being ALR. However, by far the strongest risk factor for CLR or ALR was the fact of dual resistance.

**Conclusion:**

Following a structured therapy plan based on a "test and treat" strategy, the prevalence of clopidogrel or aspirin low response can be significantly reduced and the risk of inadequate dual antiplatelet therapy minimized.

**Trial Registration:**

NCT01212302 (Clinicaltrials.gov)

## Background

Dual antiplatelet therapy with acetylsalicylic acid (ASA, aspirin) and thienopyridines is of great importance for the prevention of ischemic events in patients with atherothrombotic disease. ASA has been shown to reduce early mortality in patients with acute coronary syndromes (ACS) by about one quarter and the risk of stroke or non-fatal reinfarction by about one half [1]. The platelet inhibitory effect of ASA is caused by blocking the thromboxane mediated aggregation pathway. In addition to ASA, the thienopyridines ticlopidine, clopidogrel and prasugrel inhibit ADP-mediated platelet activation by blocking the P2Y12 adenosine diphosphate platelet receptor and reduce thrombotic and ischemic events [2,3].

Despite the proven benefits of dual antiplatelet therapy, complications did arise and it became apparent that the platelet inhibitory effect of clopidogrel and ASA was lower in 5 to 30% of the patients [4,5]. The mechanisms leading to poor response (low- or hypo-response, resistance) of clopidogrel are multifactoral, including lack of compliance and such clinical factors as diabetes mellitus [6]. Further data suggest that cytochrome polymorphism (that is, CYP 2C19) contribute to clopidogrel low response [7-9]. Clopidogrel low-responder (CLR) and ASA low-responder (ALR) bear a significantly higher risk of cardiovascular complications and especially of stent thrombosis [10-12]. A recent meta-analysis revealed that ASA resistance occurred on average in 28% of patients with a wide range from 0% to 57% depending on the methods used of varying time points, different cut-off values and variable concentrations of the stimulating agents [12]. Furthermore, ASA dosing differed in most studies. When taking a more specific approach, the incidence of ASA resistance in studies using arachidonic acid stimulation was approximately six percent [4]. The therapeutic options for clopidogrel and/or aspirin low-responders requiring dual anti-thrombotic therapy are still undetermined. Data suggest improving platelet inhibition in low-responders to clopidogrel by increasing the loading dose [13-15], and applying a higher maintenance dose [16-19] or by switching to alternative thienopyridine treatment (ticlopidine, prasugrel) [16,20,21]. Further studies suggest that the incidence of ASA low response is dose dependent [22], thus an option in treating ALR is to increase the ASA dose.

The aim of our study was to identify CLR and ALR in order to optimize antiplatelet therapy by using platelet function testing. We examined the hypothesis that a standardized therapeutic algorithm can reduce the prevalence of CLR and ALR.

## Methods

### Study population

Patients with either stable coronary artery disease (CAD) or acute coronary syndromes (ACS) following percutaneous coronary intervention (PCI) were enrolled in this prospective, single-center interventional study. They were treated initially with an ASA loading dose of 500 mg, followed by 100 mg ASA per day and a starting dose of 600 mg clopidogrel-hydrogensulfate, followed by a daily dose of 75 mg. Exclusion criteria were an abnormal platelet count in patients, severe liver disorders, current gastrointestinal disorders, a current infection, congestive heart failure or a known bleeding disorder as well as treatment with bivalirudin or glycoprotein IIb/IIIa antagonists within the last seven days. The present study was approved by the institutional ethics committee and complies with the Declaration of Helsinki. Written informed consent was obtained from all study participants before entering the study.

### Platelet function testing

The platelet function test used to monitor the antiplatelet effectiveness of clopidogrel and ASA was whole blood aggregometry (WBA, impedance aggregometry (IPA)) (Model 590, Chrono-log Corporation, Havertown, PA, USA) and was done >48 h following coronary stenting (but not more than 72 h later). Measurements were carried out within 60 to 180 minutes after drawing blood. Citrate blood (500 μl) was diluted 1:1 with 0.9% sodium chloride and preincubated for 10 minutes at 37°C in a polycarbonate cuvette. After a stable baseline had been established, the agonists (Chrono-Par, Chrono-log Corporation, Havertown, PA, USA), either adenosine diphosphate (ADP) with a final concentration of 5 μM or arachidonic acid (AA, final concentration 0.5 mmol/L) were added and the impedance (Ω) was registered after six minutes.

We prospectively defined low response in accordance to previous studies by setting a cut-off point for ADP-induced impedance exceeding 5 Ω for clopidogrel low response (CLR) [16,23] and AA-induced impedance values exceeding 0 Ω were defined as ASA low response (ALR) [24,25]. The results measured with the Chrono-log 590 aggregometer were reproducible with a variability <10%.

Kidney function was assessed using the estimated glomerular filtration rate (eGFR), calculated from serum creatinine using the Cockcroft-Gault method. Diabetes mellitus was defined as having a physician's diagnosis in the patient's history or the intake of any antidiabetic drug or insulin. Troponin positive status was defined when troponin T values exceeded 0.1 ng/mL. The PREDICT-score was calculated as previously shown, including such clinical variables as ACS, diabetes mellitus, renal failure, age >65 years and reduced left ventricular function [26].

Bleeding complications were recorded during the hospital stay or by interview when further platelet function tests had to be scheduled. Hemorrhagic complications were defined as major bleedings in the case of intracranial haemorrhage, a drop in the haemoglobin level of 3 g/dl or bleeding at the access site that required intervention or the need of blood transfusions.

### Study protocol

**Figure 1:**
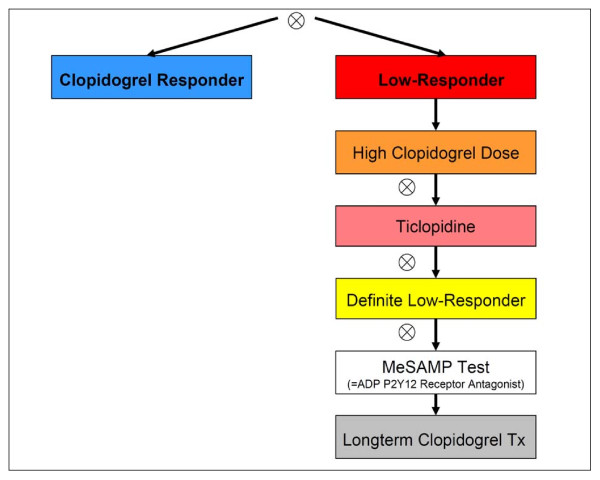
**Study plan optimizing clopidogrel treatment without prasugrel**. (Prasugrel not available or contraindicated). Platelet function assay (if treatment ineffective, next step), Tx indicates treatment; high clopidogrel dose of 150 mg daily, ticlopidine 2 × 250 mg daily.

**Figure 2:**
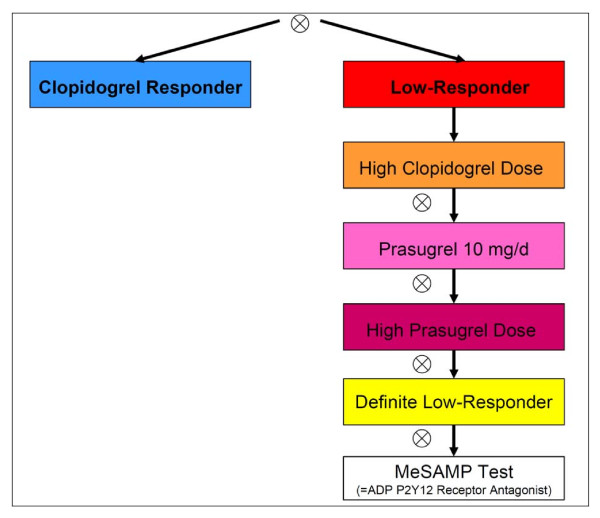
**Study plan optimizing clopidogrel treatment including prasugrel**. (Prasugrel available and not contraindicated). Platelet function assay (if treatment ineffective, next step), high clopidogrel dose of 150 mg daily, high prasugrel dose of 20 mg daily.

**Figure 3:**
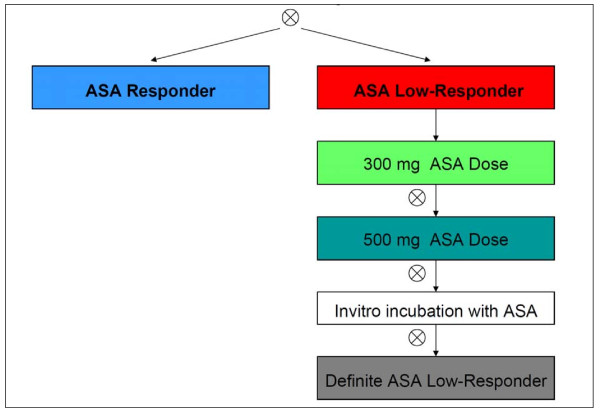
**Study plan optimizing ASA treatment**. Platelet function assay (if treatment ineffective, next step), ASA (acetylsalicylic acid, aspirin) 100 mg daily.

In the case of low response the ASA and/or clopidogrel treatment was modified in accordance to a structured therapy plan (Figures 1, 2 and 3).

The prespecified therapy plan for CLR 75 mg included the following options: as a first step, standard dose clopidogrel low responders were given another loading dose (600 mg), followed by a doubled clopidogrel maintenance dose (150 mg daily). If the measurements still did not reveal an adequate effect, the next step, as long as prasugrel was not available, was to change the ADP-inhibitory medication to ticlopidine 250 mg twice daily (after a loading dose of 500 mg was given) and again the platelet inhibitory effect was evaluated 48 h later by aggregometry. For CLR and ticlopidine low responders (TLRs), a defect of the ADP-receptor was evaluated by the selective P2Y12 ADP receptor antagonist 2-methylthioadenosine 5'-monophosphate triethylammonium salt (MeSAMP, Sigma-Aldrich, Munich, Germany) to disclose a defect at the level of the P2Y12 ADP receptor (Figure 1). When prasugrel was not available (up until April 2009) or contraindicated, CLR and TLR patients were kept on long-term treatment with high clopidogrel dosage and follow-up measurements were scheduled regularly. On including prasugrel, the therapy plan was modified as follows (Figure 2): Clopidogrel high dose low responders were switched to prasugrel (60 mg loading, 10 mg prasugrel daily) and the measurements were repeated (>48 h). In the case of prasugrel low response (PLR), the dose was increased to 20 mg prasugrel daily (once again IPA-testing was carried out >48 h later). However, in the case of increased thienopyridine treatment dosages, the time period was limited to four weeks following bare metal stenting (BMS) and six months when drug eluting stents (DES) had been used.

The therapy algorithm for ALRs included as a first step the application of another loading dose of 500 mg ASA, followed by 300 mg ASA daily (Figure 3). If these patients showed an adequate inhibitory effect in IPA testing (<1 ohm of AA induced platelet aggregation) 48 hours later, they were defined as ASA "high maintenance dose" responders. On detecting an insufficient inhibitory effect, the ASA dose was further increased to 500 mg ASA daily ("maximum dose ASA" response if adequate IPA results existed 48 h later). To rule out pharmacokinetic ASA resistance, the blood samples were incubated with 500 mg ASA ("ASA test"). Patients without a sufficient platelet inhibitory effect on a dose of 500 mg ASA daily were defined as "ASA definite low responder".

### Statistical analysis

Univariate analysis was performed with X^2 ^testing for categorical variables and a *t*-test (Mann-Whitney *U *test for non-normal distributions) for continuous variables. The odds ratios for the association between predictors (independent variables) and ASA or clopidogrel low response status were expressed in corresponding two-sided 95% confidence intervals (CIs) and *P*-values.

Sample size calculation for the present study was based on the assumption that the incidence of clopidogrel low response was at least 20% and ASA low response 10%. Choosing a power of 97.5% and a two-sided value of 0.05, an overall sample size was required of at least 400 patients. To compensate for a possible loss of follow-up, we aimed for inclusion of 500 patients.

All statistical calculations were performed using SAS, version 9.1 (SAS Institute Inc., Cary, NC, USA) and SPSS 18 (SPSS, Chicago, IL, USA).

## Results

### Clinical and demographic data

**Figure 4:**
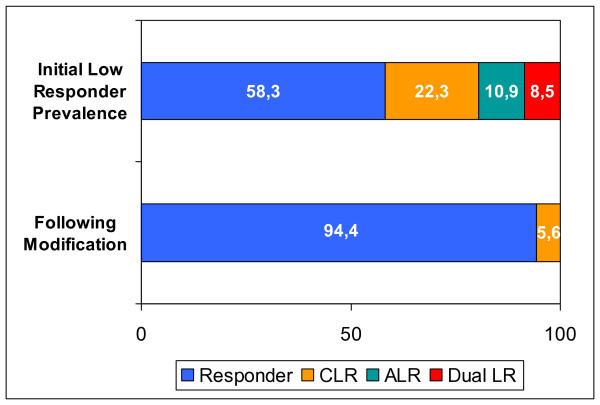
**Results of the entire study group**. Prevalence of ALR, CLR and Dual LR before and after modification according to the therapy algorithm (without prasugrel) Percentage of dual responder (Responder), clopidogrel low responder (CLR), ASA low responder (ALR) and dual low responder (Dual LR) before and after optimization of antiplatelet therapy. Note: By applying the therapy algorithm (without prasugrel), the low responder rate can be reduced absolute by 36.1% (relative -86.6%) and including prasugrel low response was eliminated.

A total of 504 patients treated with PCI participated in the study. The prevalence of dual responders to antiplatelet therapy with ASA and clopidogrel was 58.3%, isolated CLR 22.3%, isolated ALR 10.9% and the rate of dual low response was 8.5% (Figure 4).

The mean age was 64.3 ± 11.7 years, 30.5% were females and the majority of patients (67.8%) had an acute coronary syndrome (ACS). The patients took multiple co-medications (number of drugs 7.5 +/- 2.4) with a high rate of ACE-inhibitors (87.1%), ß-blockers (88.0%), statins (79.1%) and proton pump inhibitors (PPIs; 42.3%). Clinical and baseline characteristics of the study population are shown in Tables 1 and 2. During the hospital stay and in the case of follow-up visits due to repeated platelet function measurements, we did not observe any significant differences between the treatment groups in regard to hemorrhagic complications (major bleeding events) and the need for blood transfusions.

**Table 1:** Baseline Demographic and Clinical Characteristics Comparing Clopidogrel Low Responder and Responder

Variable	Clopidogrel Low-Responder	Clopidogrel Responder	OR (95% CI)	p-Value
No. (%)	155 (30.8)	349 (69.2)		

Female gender (%)	54 (34.8)	101 (28.9)	1.31 (0.88-1.97)	n.s. (0.21)

Age (years)	64.5 (11.5)	64.4 (11.7)		n.s. (0.93)

BMI (kg/m^2^)	28.06 (5.00)	27.81 (4.20)		n.s. (0.56)

EF (%)	52.82 (12.50)	54.23 (10.50)		n.s. (0.19)

Acute coronary syndrome	122 (78.7)	214 (61.3)	2.33 (1.50-3.63)	<0.001

Arterial hypertension	124 (80.0)	282 (80.8)	0.95 (0.59-1.53)	n.s. (0.90)

Diabetes mellitus	74 (47.7)	124 (35.5)	1.66 (1.13-2.43)	0.01

Lipid disorder	95 (61.3)	211 (60.5)	1.04 (0.70-1.53)	n.s. (0.92)

Cigarette smoking	68 (43.9)	157 (45.0)	0.96 (0.65-1.40)	n.s. (0.85)

Familial CAD disposition	33 (21.3)	63 (18.1)	1.23 (0.77-1.97)	n.s. (0.39)


ASA 100 mg	153 (98.7)	349 (100)	0.09 (0.01-1.84)	n.s. (0.09)

Beta-Blockers	138 (89.0)	302 (86.5)	1.26 (0.70-2.28)	n.s. (0.47)

Nitrates	24 (15.5)	72 (20.6)	0.70 (0.42-1.17)	n.s. (0.22)

Calcium-channel blockers	49 (31.6)	101 (28.9)	1.14 (0.75-1.71)	n.s. (0.60)

ACE Inhibitors	133 (85.8)	301 (86.2)	0.96 (0.56-1.66)	n.s. (0.89)

Diuretics	73 (47.1)	138 (39.5)	1.36 (0.93-1.99)	n.s. (0.12)

Statins	125 (80.6)	269 (77.1)	1.24 (0.77-1.98)	n.s. (0.41)

Proton pump inhibitors	62 (40.0)	151 (43.3)	0.87 (0.59-1.29)	n.s. (0.56)

Number of drugs	7.69 (2.30)	7.36 (2.30)		n.s. (0.14)

WBC (×10^9^/l)	9.22 (4.17)	8.71 (6.10)		n.s. (0.34)

Hemoglobin (g/dl)	14.12 (1.60)	14.28 (1.90)		n.s. (0.36)

Platelet count (×10^9^/l)	230.34 (69.62)	212.00 (87.97)		0.02

Troponin positive (%)	35 (22.6)	37 (10.6)	2.46 (1.48-4.09)	<0.001

CK max (U/l)	428.85 (1246.60)	391.61 (783.70)		n.s. (0.68)

Total cholesterol (mg/dl)	203.70 (57.30)	204.37 (50.10)		n.s. (0.89)

CRP (mg/l)	11.47 (20.70)	7.34 (15.40)		0.01

HbA_1C _(%)	6.71 (1.60)	6.42 (1.30)		0.03

D-dimer (mg/l)	0.57 (0.50)	0.57 (0.40)		n.s. (1.00)

Serum creatinine (mg/dl)	1.06 (0.60)	1.04 (0.30)		n.s. (0.62)

GFR (MDRD) (ml/min)	76.34 (24.20)	75.87 (20.90)		n.s. (0.82)

PREDICT-score	3.38 (2.0)	2.48 (1.90)		<0.001

ASA low-response	44 (28.4)	31 (8.9)	4.07 (2.45-6.76)	<0.001

Data presented are mean ± SD and count (no.) or percentage (%). Calculation of p-values was done by unpaired t-test or chi-square test, comparing low-responder versus responder. OR = odds ratio; CI = confidence interval; n.s. = not significant.

BMI = body mass index, EF = ejection fraction, CAD = coronary artery disease, ASA = acetylsalicylic acid (aspirin), ACE = angiotensin-converting enzyme, WBC = white blood cells, CK = creatine kinase, CRP = C-reactive protein, HbA_1C _= hemoglobin A1c, GFR = glomerular filtration rate.

**Table 2:** Baseline Demographic and Clinical Characteristics Comparing ASA Low Responder and Responder

Variable	ASA Low-Responder	ASA Responder	OR (95% CI)	p-Value
No. (%)	78 (19.4)	325 (80.6)		

Female gender (%)	19 (24.4)	101 (31.1)	0.71 (0.40-1.26)	n.s. (0.27)

Age (years)	63.80 (12.90)	64.30 (11.70)		n.s. (0.74)

BMI (kg/m^2^)	28.17 (5.10)	27.81 (4.30)		n.s. (0.52)

EF (%)	52.78 (13.90)	54.35 (11.10)		n.s. (0.29)

Acute coronary syndrome	61 (78.2)	211 (64.9)	1.94 (1.08-3.48)	0.03

Arterial hypertension	62 (79.5)	258 (79.4)	1.01 (0.54-1.86)	n.s. (1.0)

Diabetes mellitus	38 (48.7)	113 (34.8)	1.78 (1.08-2.94)	0.03

Lipid disorder	44 (56.4)	193 (59.4)	0.89 (0.54-1.46)	n.s. (0.70)

Cigarette smoking	33 (42.3)	148 (45.5)	0.88 (0.53-1.45)	n.s. (0.62)

Familial CAD disposition	10 (12.8)	68 (20.9)	0.56 (0.27-1.14)	n.s. (0.11)

Beta-Blockers	71 (91.0)	279 (85.8)	1.67 (0.72-3.86)	n.s. (0.27)

Nitrates	8 (10.3)	71 (21.8)	0.41 (0.19-0.89)	0.03

Calcium-channel blockers	25 (32.0)	90 (27.7)	1.23 (0.72-2.10)	n.s. (0.49)

ACE Inhibitors	67 (85.9)	276 (84.9)	1.08 (0.53-2.19)	n.s. (1.0)

Diuretics	39 (50.0)	124 (38.2)	1.62 (0.99-2.67)	n.s. (0.07)

Statins	58 (74.4)	257 (79.1)	0.77 (0.43-1.36)	n.s. (0.36)

Proton pump inhibitors	29 (37.2)	147 (45.2)	0.72 (0.43-1.19)	n.s. (0.21)

Number of drugs (n ± SD)	7.50 (2.80)	7.48 (2.20)	1.67 (0.72-3.86)	n.s. (0.95)

WBC (×10^9^/l)	9.80 (4.10)	8.70 (5.40)		0.09

Hemoglobin (g/dl)	14.71 (2.40)	14.07 (1.50)		0.003

Platelet count (×10^9^/l)	250.33 (10.22)	205.80 (63.69)		<0.001

Troponin positive (%)	34 (43.6)	53 (16.3)	3.97 (2.32-6.78)	<0.001

CK max (U/l)	478.15 (1508.40)	529.77 (638.70)		n.s. (0.64)

Total cholesterol (mg/dl)	205.63 (60.60)	202.14 (51.30)		n.s. (0.60)

CRP (mg/l)	12.26 (21.40)	7.22 (15.40)		0.02

HbA_1C _(%)	6.88 (1.70)	6.41 (1.30)		0.007

D-dimer (mg/l)	0.78 (0.60)	1.02 (2.70)		n.s. (0.44)

Serum creatinine (mg/dl)	1.14 (0.80)	1.02 (0.30)		0.03

GFR (MDRD) (ml/min)	81.50 (59.70)	78.16 (22.30)		n.s. (0.42)

PREDICT score	3.39 (2.20)	2.55 (1.80)		<0.001

Clopidogrel low-response	44 (56.4)	74 (22.8)	4.39 (2.62-7.36)	<0.001

Data presented are mean ± SD and count (no.) or percentage (%). Calculation of p-values was done by unpaired t-test or chi-square test, comparing low-responder versus responder. OR = odds ratio; CI = confidence interval; n.s. = not significant.

ASA = acetylsalicylic acid (aspirin), BMI = body mass index, EF = ejection fraction, CAD = coronary artery disease, ACE = angiotensin-converting enzyme, WBC = white blood cells, CK = creatine kinase, CRP = C-reactive protein, HbA_1C _= hemoglobin A1c, GFR = glomerular filtration rate.

### Clopidogrel low response and the effect of therapy modification

**Figure 5:**
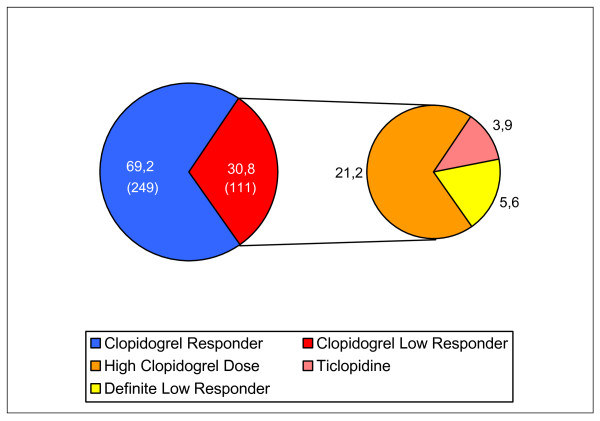
**Results of clopidogrel low-response (CLR) following therapy modification without prasugrel**. The left section of the figure shows the prevalence in % (numbers) of clopidogrel responder and clopidogrel low responder (CLR). In the right section are the results after therapy modification according to the therapy algorithm for CLR. Treatment options used: clopidogrel responder (75 mg daily), high clopidogrel dose (150 mg daily), ticlopidine (250 mg twice daily).

We identified 30.8% (n = 155) of the patients to be CLR. By increasing the clopidogrel maintenance dose from 75 mg to 150 mg daily, the majority (69.0%) of the CLRs were treated effectively. When prasugrel was not available or contraindicated, patients without an adequate response despite the high clopidogrel maintenance dose were switched to ticlopidine. By doing this, 12.7% of the high dose CLR attained a sufficient inhibition of ADP-induced platelet aggregation. When the patients still had an inadequate response (5.6% of the whole study group) they were kept on a high clopidogrel maintenance dose and long-term follow-up measurements were scheduled (Figure 5). On this dosage, 3 out of 13 definite CLR patients gained an adequate response in the following weeks.

**Figure 6:**
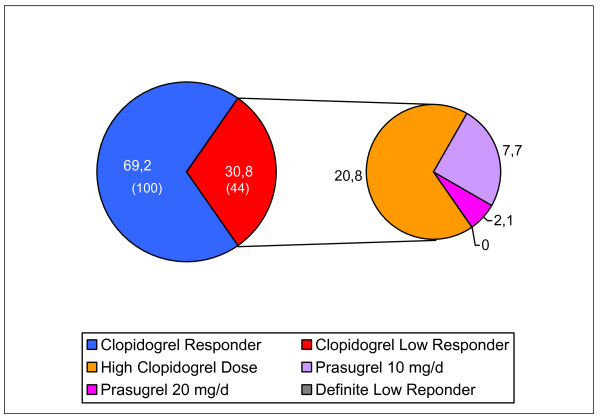
**Results of clopidogrel low-response (CLR) following therapy modification including prasugrel**. The left section of the figure shows the prevalence in % (numbers) of clopidogrel responder and clopidogrel low responder (CLR). In the right section are the results after therapy modification according to the therapy algorithm for CLR including prasugrel. Clopidogrel responder were treated with 75 mg daily and the high clopidogrel dose was 150 mg daily. Definite low responder were low responder to either clopidogrel high dose or prasugrel high dose.

When applying the treatment plan and including prasugrel we made the following findings: of the CLR on a high maintenance dose, 92% were effectively treated with prasugrel 10 mg daily. The prasugrel dose had to be adjusted to 20 mg daily in only three patients and finally all patients gained an adequate platelet inhibitory effect (Figure 6).

### ASA low response and effect of therapy modification

**Figure 7:**
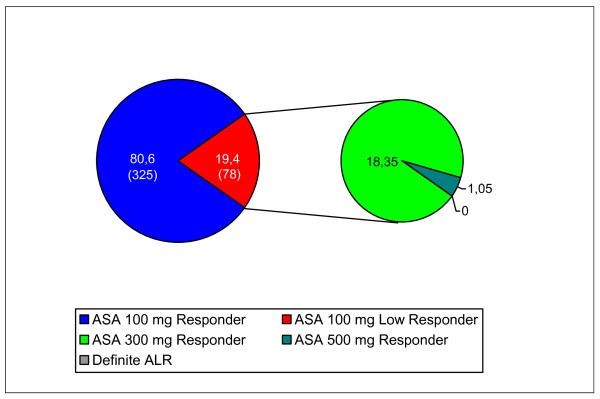
**Results of ASA low response (ALR) before and after therapy modification**. The left section of the figure shows the prevalence in % (numbers) of ASA responder and ASA low responder (ALR). In the right section are the results after therapy adjustment of ASA dose according to the therapy algorithm for ALR.

Measurements of ASA effectiveness were taken of 403 patients. The rate of ASA low responsiveness was 19.4% (78 patients). Loss of follow-up data in 22 patients was caused by a transfer to coronary artery bypass grafts (CABG), by the patients having been discharged or proven to be ineligible for treatment in accordance with the study plan (for example gastrointestinal reasons). Of the ASA low responders who were optimized according to the therapy algorithm, 94.6% (n = 53) were effectively treated by using a 300 mg ASA maintenance dose. By increasing the ASA dose to 500 mg daily all remaining ALRs finally became ASA responders (Figure 7). In conclusion, guided by platelet function test, ASA resistance was eliminated after individual dosage adjustments were made according to the treatment scheme.

### Identifying risk factors for CLR or ALR

The analysis of the demographic and clinical data of CLR revealed that the following factors influenced the incidence of clopidogrel low response (Table 1): as is true of ALR, acute coronary syndromes, and in this context positive troponin values as well, were associated with CLR. The incidence of diabetes mellitus and elevated HbA_1C _values were more frequently detected in CLR. Other significantly elevated laboratory values were the platelet count and C-reactive protein. We identified the PREDICT- score to be a strong and significant marker for the risk of being CLR (*P *< 0.001). But by far the strongest risk factor for CLR is an ASA low response status (OR 4.07 (2.45 to 6.76).

The following factors increased the risk of ASA low response (Table 2): Patients with acute coronary syndromes ran a significantly higher risk of being ALR (OR 1.94 (1.08 to 3.48)). Accordingly, a positive troponin status was a strong predictor for ALR (OR 3.97 (2.32 to 6.78)). Other clinical factors related to ALR were diabetes mellitus (OR 1.78 (1.08 to 2.94)) and elevated HbA_1C _values (*P *= 0.007). Patients without nitrate medication ran an increased risk of being ALR, and the same held true for elevated hemoglobin, platelet count and serum creatinine values. An interesting finding was that in the group of ALR, C-reactive protein values as a marker of inflammation were significantly elevated (*P *= 0.02). As is the case with CLR on calculating the PREDICT-score, a strong correlation existed between a high PREDICT-score and ALR. The strongest factor for ALR, however, was the existence of CLR (OR 4.39 (2.62 to 7.36)).

### Double low response

Out of the total study group we found 43 patients (8.5%) to be dual low responders (ALR and CLR). As is true for either ALR or CLR, risk factors for dual low response were a high PREDICT-score (*P *= 0.002), acute coronary syndrome (*P *= 0.006), patients with positive troponin values (p < 0.001) and elevated creatine kinase values (*P *= 0.02) (Additional File 1. Supplement Table 1). In addition we detected more patients with dual low response when no nitrate medication was given (*P *= 0.02). Furthermore, risk factors for dual low response were significantly elevated serum creatinine (*P *= 0.02) and C-reactive protein values (*P *= 0.006).

### Evaluation of an ADP-receptor defect or ASA pharmacodynamic problem

In high dose CLR, MeSAMP testing was carried out and did not reveal any case of ADP-receptor problem in our study. Furthermore, we detected that all ALR showed an adequate inhibitory effect of AA-induced platelet aggregation when the blood samples were incubated *in vitro *with ASA (ASA-test).

## Discussion

The present study demonstrates that dual antiplatelet therapy using ASA and clopidogrel can be significantly improved when using a tailored "test and treat" approach. Our data revealed that following a structured therapy plan based on platelet function testing, ASA low response can be overcome by stepwise increasing the ASA maintenance dose. Also, the prevalence of clopidogrel hypo-/low-response can be significantly decreased by 86.6% by dose escalation or switch to ticlopidine. When prasugrel was included in the therapy algorithm, thienopyridine resistance was able to be eliminated.

Antiplatelet agents provide the cornerstone of medical treatment in cardiovascular medicine. Despite the proven benefit of preventing stent thrombosis following PCI and stent implantation, dual antiplatelet therapy with ASA and thienopyridines (ticlopidine or clopidogrel) is limited by the variability in the antiplatelet response [4,5]. Data have linked therapeutic failure (resistance, low- or hypo-responsiveness) to antiplatelet therapy to an increased risk of cardiovascular complications including stent thrombosis [10]. Compared to clopidogrel resistance, ASA resistance has so far received little attention in interventional cardiology. But as is true of clopidogrel, the response to ASA treatment is variable and resistance to ASA is linked to an increase in the major adverse cardiac event (MACE) rate [12]. In our study we found 30.8% of the patients to be CLR and 19.4% to be ALR. Of these patients 8.5% were dual low responders. With the need for more effective and sustained antiplatelet therapy to reduce cardiovascular morbidity, it is worthwhile to evaluate the effect of dual antiplatelet therapy using medications, ASA and thienopyridines, in order to optimize the effectiveness of the platelet inhibitory response. With the use of the presented therapeutic algorithm we were able to overcome resistance to ASA and CLR either by 86.6% (without prasugrel) or even to overcome it when prasugrel was included. Our structured therapy plan for optimizing dual antiplatelet therapy does not depend on a specific platelet test. We chose whole blood platelet testing because of several advantages it provided: compared to other assays, the method used is inexpensive, easy to perform and requires only 10 minutes [27]. The multiple electrode aggregometry (MEA) test system uses a similar technique [4,27]. However, other methods to monitor the response of antiplatelet medication can also be used to guide the therapy modifications according to the therapy algorithm presented here.

### Improving CLR

What are the options on how to treat clopidogrel low responders? Data suggest improving platelet inhibition in low-responders to clopidogrel by using alternative treatment strategies, including either an increase in the loading dose [13-15] or the use of a higher maintenance dose [16-19], or by switching to alternative thienopyridine treatment (ticlopidine, prasugrel) [16,20,21]. In addition, new platelet inhibitors are an alternative, such as ticagrelor or thrombin receptor antagonists and these agents will be introduced in the near future [28,29].

Data demonstrated that adjusting the clopidogrel loading dose according to platelet function testing translates to a significantly improved short-term outcome following PCI [30]. The RELOAD study reveals that a new clopidogrel loading dose in patients treated long term with 75 mg clopidogrel daily, improves platelet inhibition and this in turn reduces low response [31].

Regarding the maintenance therapy, studies have demonstrated that 150 mg clopidogrel lead to a greater inhibition of platelet aggregation than a 75 mg daily dose [17,18]. The ACC Practice Guidelines of 2005 recommend (Class IIb) increasing the dose of clopidogrel to 150 mg per day if less than 50% inhibition of platelet aggregation is apparent [32]. The CURRENT-OASIS-7 study evaluated the effect of 600 mg loading dosage and then doubling the maintenance dose (150 mg) of clopidogrel in ACS patients as compared to standard therapy (300 mg, followed by 75 mg) [19]. In this study, all patients on a higher clopidogrel dosage had lower MACE rates. However, with this unselected approach involving a higher clopidogrel dosage irrespective of the antiplatelet effect, the study group observed a higher rate of major bleeding events according to the "CURRENT" definitions [19]. Using a higher clopidogrel dose only in risk patients (CLR) would (theoretically) avoid this unnecessary overtreatment. Further studies will have to prove if this approach of platelet function testing will lead to an improvement of antiplatelet therapy, thus providing better clinical results [33]. Given the options to overcome clopidogrel resistance, we optimized the antiplatelet therapy according to a pre-defined therapy plan guided by platelet function testing. In a first step we combined both: In the case of CLR, an additional loading dose of 600 mg clopidogrel was given and the maintenance dose was increased to 150 mg daily. On this dosage, the majority of patients (69%) was able to obtain an adequate inhibitory response to ADP-induced aggregation.

Another option to overcome CLR is to switch the medication to other thienopyridines. As long as contraindications for prasugrel apply (history of stroke, age >75 years, <65 kg) or prasugrel was not available, ticlopidine was used in patients with high dose CLR. We, and others, have shown that ticlopidine with a partly different metabolism is an alternative in treating CLR patients [16,20]. With the use of ticlopidine instead of clopidogrel, our study demonstrates that 12.7% of the remaining CLR can be effectively treated despite the use of high dose clopidogrel. The reason for this could be the partially different metabolism of ticlopidine as compared to clopidogrel, with CYP2B6 and CYP2C19 contributing mainly to the activation of ticlopidine [2]. However, it has to be kept in mind that the inferior safety profile of ticlopidine involves the risk of such side effects as neutropenia in roughly 2.4% of patients [5]. We, therefore, preferred using clopidogrel in higher maintenance dosages instead of ticlopidine.

When the third generation thienopyridine prasugrel was included in the therapy algorithm, 92% of the high dose CLRs were treated effectively with a standard dose of prasugrel and the remaining 8% showed an adequate platelet inhibitory response on 20 mg prasugrel. Our study revealed that when including prasugrel, we were able to eliminate thienopyridine resistance. Compared to clopidogrel, prasugrel has the advantage of providing a less variable, but more effective and faster antiplatelet response due to a more effective metabolism [2,29]. In contrast to the two-step metabolism of clopidogrel, prasugrel is transformed by a single CYP-dependent oxidative step into its active metabolite [2]. As a result prasugrel achieves a 10 times higher level of the active metabolite than clopidogrel [2,29].

### Improving ALR

Compared to clopidogrel, the data on the clinical impact of ASA low response in patients treated with dual antiplatelet medication are limited as most studies focus primarily on identifying and treating clopidogrel resistance. Nevertheless, a meta-analysis revealed that patients who are resistant to ASA are at a greater risk of clinically important cardiovascular morbidity than patients who are sensitive to ASA [12]. The ALR prevalence depends on the test method and agonist used, as well as on the clinical setting (high proportion of patients with ACS in our study (67.8%)), ASA dose and cut-off definition [4,5]. As is true of clopidogrel treatment, the influence of non-compliance can attribute to the prevalence of ASA low response [4]. However, as our measurements were done early after PCI during the hospital stay, we do not assume that a high proportion of non compliance as a cause for ALR or CLR. In our study the prevalence of ALR was 19.4%. As we were able to eliminate ASA low response by stepwise increasing the ASA dose, other investigators found similar results: In the ASPECT study with stable CAD, the ALR rate was 1.6% on 81 mg ASA daily, but it was no longer detected when using 325 mg ASA [22]. Similar findings were published by another working group, with an ALR prevalence of 2% in 700 patients, which was caused by non-compliance or underdosing [34]. Recently the randomized CURRENT-OASIS-7 study has revealed that the efficacy of ASA to reduce the MACE rate as well as safety (bleeding events) did not differ between high dose (300 to 325 mg) and low dose ASA (75 to 100 mg) [19]. However, it must also be taken into consideration that in other studies an increase of ASA doses was associated with an increase in adverse events, such as haemorrhages [3-5].

### Dual resistance of antiplatelet agents

A special group to be considered are patients with dual antiplatelet resistance as these patients bear the greatest risk of major adverse events, such as stent thrombosis. In accordance with previous studies we identified a prevalence of 8.5% to be dual low responders. An analysis of the RECLOSE trial cohort showed a prevalence of 6% with dual resistance to ASA and clopidogrel [35]. This dual non-responsiveness was an independent risk factor and led to markedly higher rates of DES thrombosis (11.1%) as compared to isolated ASA (2.3%) or clopidogrel non-responsiveness (2.2%) [35]. Other data revealed a prevalence of 10.4% dual low response and suggest a high cardiovascular risk after PCI for these patients with the need for intensified antiplatelet therapy and follow-up [23]. As a consequence not only CLR should be identified to determine the patients' risk, but ALR is relevant to the clinical outcome as well.

A single approach focussing on just one option to overcome resistance to antiplatelet drugs has a limited potential [36]. Therefore, a structured stepwise therapy algorithm to individualize antiplatelet therapy is preferable. This is a superior option compared to intensifying the antiplatelet therapy for all patients regardless of the degree of platelet inhibition as was done in the CURRRENT-OASIS-7 study as this involves the risk of increased adverse bleeding events [19].

### Factors influencing low response

In accordance with other studies we identified risk markers for ALR and CLR. These clinical and demographic characteristics are mainly comorbidities and concomitant medication [29]. Analysis of the present study revealed that ACS and elevated troponin values were strong risk factors for both CLR and ALR. It is known that ACS patients are at risk due to an increased residual platelet activity in clopidogrel and ASA-treated patients as compared to those with a stable coronary artery disease [26,37]. Especially patients with ACS might therefore benefit from an alternative or intensified antiplatelet regimen as we have shown in our study. Other known risk factors for CLR and ALR, which we detected, were diabetes mellitus and in this context elevated HbA_1C _values [26,38]. Interestingly, we found that C-reactive protein as a marker of inflammation as well as platelet count was significantly elevated in CLR and ALR in our study. Furthermore, the data of the present study suggest that concomitant medication with nitrates decreases the risk of ALR. In contrast to other data, we did not find an association with CLR status in regard to the BMI or co-medication with proton pump inhibitors and calcium antagonists [29]. In summary, when taking all the characteristics into consideration (Tables 1 and 2), the most important risk for low response proved to be a low response to ASA or clopidogrel. The value of the PREDICT-score was confirmed (ACS, reduced LV-function, diabetes mellitus, creatinine >1.5 mg/dL, age >65 years) [26]. Therefore, if platelet function assays are not available, the use of the PREDICT-score can be used to identify risk patients. However, it is important to note that even if it is worthwhile to identify such risk factors for low response as demographic and clinical variables or in genotyping patients (CYP2C19*2 polymorphism), analysis of the EXCELSIOR-study revealed only a limited predictive value for this and moreover, this analysis suggests that platelet function analysis is much more useful [37].

The reasons for resistance to antiplatelet medication with ASA and clopidogrel are clinical, cellular and genetic factors [6,29]. One potential mechanism which causes a diminished response to thienopyridine medication is an ADP receptor defect. Therefore we ruled out in the case of definite CLR by MeSAMP testing (specific ADP receptor antagonist) an ADP receptor defect as a cause of clopidogrel resistance. Regarding ASA treatment, we found all ALR to have a pharmacokinetic resistance as *in vitro *incubation with ASA revealed in all ALR patients an adequate platelet inhibitory effect.

## Limitations

Our observational, non-randomized study suggests that antiplatelet therapy can be significantly improved. The cut-off values and the method used have limitations as no large scale clinical trials have yet prospectively linked the measurements to adverse clinical outcomes. In this context, an approach to define cut-off values for different platelet function assays was recently presented [39]. Prospective randomized trials are needed to prove the clinical benefits of adapting the dosing of clopidogrel or switching to alternative compounds in high-risk patients with impaired antiplatelet effectiveness according to the result of platelet function assays. Future studies will have to further evaluate if the strategy to improve biochemical response leads to significantly improved MACE rates without causing an increased risk of bleeding.

## Conclusions

Following a structured therapy plan based on a "test and treat" strategy, the prevalence of clopidogrel or aspirin low response can be significantly reduced and the risk of inadequate dual antiplatelet therapy minimized. Thus, an individual tailored therapy can significantly improve the effect of antiplatelet treatment in a majority of patients after coronary stenting and eliminate resistance to antiplatelet therapy.

## Abbreviations

AA: arachidonic acid; ACE: angiotensin converting enzyme; ACS: acute coronary syndrome; ADP: adenosine diphosphate; ALR: ASA low responder (response); ASA: acetylsalicylic acid (aspirin); BMI: body mass index; BMS: bare metal stent; CABG: coronary artery bypasses graft; CAD: coronary artery disease; CI: confidence interval; CLR: clopidogrel low responder (response); CYP: cytochrome P450; DES: drug eluting stent; EF: ejection fraction; eGFR: estimated glomerular filtration rate' HbA_1C_: glycated haemoglobin A1c; IPA: impedance aggregometry; MACE: major adverse cardiac event; MEA: multiple electrode aggregometry; MeSAMP: 2-methylthioadenosine 5'-monophosphate triethylammonium salt; PCI: percutaneous coronary intervention; PLR: prasugrel low responder (reponse); PPI: proton pump inhibitors; TLR: ticlopidine low responder (response); WBA: whole blood aggregometry;

## Competing interests

The authors declare that they have no competing interests.

## Authors' contributions

HN drafted the manuscript, supervised the data collection and was substantially involved in the study design and statistical analysis. SL, AE, JCK and AFCK carried out the platelet function studies and were involved in the patient and data collection. SL, AE and JCK were also involved in the study design. AK and FP supervised the platelet function studies and were involved in the data and patient collection. HGE supervised the statistical analysis and helped draft the manuscript. AM participated in the study design, supervised the patient collection and assisted in the coordination of the entire study. All authors have read and approved the final manuscript.

## Pre-publication history

The pre-publication history for this paper can be accessed here:

http://www.biomedcentral.com/1741-7015/9/3/prepub

## Supplementary Material

Additional file 1 **Supplement table**. Baseline demographic and clinical characteristics of clopidogrel and ASA treated patients compared to dual low responder. Click here for file
