# Supplementary material for: Tailored antiplatelet therapy can overcome clopidogrel and aspirin resistance - The BOchum CLopidogrel and Aspirin Plan (BOCLA-Plan) to improve antiplatelet therapy
Source: BMC Med. 2011 Jan 12;9:3. doi: 10.1186/1741-7015-9-3 (PMC3033359; doi:10.1186/1741-7015-9-3)
Supplement: Additional file 1 — Supplement table. Baseline demographic and clinical characteristics of clopidogrel and ASA treated patients compared to dual low responder. [file 1741-7015-9-3-S1.DOC]

**Supplement Table:** Baseline Demographic and Clinical Characteristics of Clopidogrel and ASA Treated Patients Compared to Dual Low Responders

| **Variable** | **Study group** | **Dual Low Responder** | **p-Value** |
| --- | --- | --- | --- |
| No. (%) | 461 | 43 |  |
| Female gender (% per group) | 145 (31.50) | 9 (20.90) | 0.17 |
| Age (years) | 64.50 (11.50) | 63.90 (13.10) | 0.75 |
| BMI (kg/m²) | 27.87 (4.40) | 28.22 (5.40) | 0.63 |
| EF (%) | 54.01 (10.90) | 51.41 (13.90) | 0.16 |
|  | | | |
| Acute coronary syndrome | 298 (65.21) | 37 (86.05) | **0.0060** |
| Arterial hypertension | 371 (82.44) | 35 (83.33) | 0.83 |
| Diabetes mellitus | 176 (38.51) | 21 (48.84) | 0.19 |
| Lipid disorder | 281 (62.58) | 24 (57.14) | 0.51 |
| Cigarette smoking | 202 (44.99) | 22 (52.38) | 0.42 |
| Familial CAD disposition | 90 (20.00) | 5 (11.90) | 0.30 |
|  | | | |
| ASA 100 mg | 461 (100) | 43 (100) | 1 |
| Beta-Blockers | 400 (87.53) | 39 (90.70) | 0.81 |
| Nitrates | 94 (20.57) | 2 (4.65) | **0.0079** |
| Calcium-channel blockers | 137 (29.98) | 13 (30.23) | 1 |
| ACE Inhibitors | 395 (86.43) | 38 (88.37) | 1 |
| Diuretics | 188 (41.14) | 23 (53.49) | 0.15 |
| Statins | 357 (78.12) | 36 (83.72) | 0.44 |
| PPI | 195 (42.76) | 17 (39.53) | 0.75 |
| Number of drugs | 7.48 (2.30) | 7.63 (2.50) | 0.77 |
|  | | | |
| WBC (< 1000/m³) | 8533.03 (5436.90) | 10390.00 (4195.00) | 0.11 |
| Hemoglobin (g/dl) | 14.20 (1.80) | 14.38 (1.80) | 0.65 |
| Platelet count (x10^9^/l) | 213374 (80694) | 246833 (81379) | **0.06** |
| Troponine pos (%) | 51 (43.59) | 20 (86.96) | **0.0001** |
| CK max (U/l) | 502.18 (715.50) | 822.14 (1689.50) | 0.12 |
| Total Cholesterol (mg/dl) | 205.39 (52.50) | 190.49 (59.10) | 0.11 |
| CRP (mg/l) | 7.34 (14.90) | 18.25 (27.30) | **0.0061** |
| HbA_1C_ (%) | 6.50 (1.30) | 6.66 (1.80) | 0.54 |
| D-dimer (mg/l) | 0.52 (0.30) | 0.77 (0.60) | 0.13 |
| Creatinine ± SD | 1.04 (0.30) | 1.20 (1.00) | **0.0157** |
| GFR (MDRD) ± SD | 76.09 (21.70) | 75.18 (25.00) | 0.85 |
|  | | | |
| PREDICT-score ± SD | 2.66 (1.90) | 3.68 (2.00) | **0.0017** |

Data presented are mean ± SD or n (%). Calculation of p-values was done by unpaired t-test or Chi-Quadrat test, comparing low-responder versus study group.

ASA = acetylsalicylic acid (aspirin), BMI = body mass index, EF = ejection fraction, CAD = coronary artery disease, ASA = acetylsalicylic acid, ACE = angiotensin-converting enzyme, WBC = white blood cells, CK = creatine kinase, CRP = C-reactive proteine, HbA_1C_ = hemoglobin A1c, GFR = glomerular filtration rate.
